# Sun Exposure in Pediatric Age: Perspective of Caregivers

**DOI:** 10.3390/children8111019

**Published:** 2021-11-06

**Authors:** Mafalda Salvado, Ana Fraga, Diogo Luís Marques, Ivan Miguel Pires, Carmo Coelho Gonçalves, Nádia Mendes Silva

**Affiliations:** 1 Unidade de Saúde Familiar Cidade do Lis, Centro de Saúde Dr. Gorjão Henriques, 2410-272 Leiria, Portugal; nrsilva@arscentro.min-saude.pt; 2 Serviço de Pediatria, Centro Hospitalar de Leiria, 2410-197 Leiria, Portugal; ana.fraga.oliveira@chleiria.min-saude.pt; 3 Department of Sport Sciences, University of Beira Interior, 6201-001 Covilhã, Portugal; diogo.marques@ubi.pt; 4 Instituto de Telecomunicações, Universidade da Beira Interior, 6200-001 Covilhã, Portugal; impires@it.ubi.pt; 5 Escola de Ciências e Tecnologia, University of Trás-os-Montes e Alto Douro, Quinta de Prados, 5001-801 Vila Real, Portugal; 6 Unidade de Saúde Familiar Vitrius, Centro de Saúde da Marinha Grande, 2430-269 Marinha Grande, Portugal; mcgoncalves@arscentro.min-saude.pt

**Keywords:** sun exposure, sun protection, pediatric age, children, adolescents, caregivers, health promotion

## Abstract

Excessive sun exposure during childhood increases the risk of skin cancer. This study characterized the knowledge and attitudes of caregivers regarding exposure and sun protection of children and adolescents. One hundred and ninety-eight caregivers (38.5 ± 8.0 years) who resorted to the consultations of a Pediatrics Service or a Family Health Unit answered a questionnaire. The age of children/adolescents was 6.5 ± 5.1 years. On average, caregivers presented a high level of knowledge regarding exposure and sun protection. One hundred and twenty-six caregivers indicated that they obtained more information about sun protection on social communication, and 66% considered the information provided by healthcare professionals to be enough. One-hundred and fifteen caregivers reported that the child/adolescent was more exposed to the sun after 4 a.m., and 88% reported applying sunscreen on the beach/pool and outdoor activities. Fifty-seven percent of caregivers renewed sunscreen application on the child/adolescent every 2 h, and 94% applied a sun protection factor ≥ 50 in the child/adolescent. There was a significant association (*p* < 0.001) between education level and caregivers’ self-knowledge about sun protection (the higher the education, the higher the knowledge), and between the knowledge of the hour of sun exposure avoidance and the time when the child/adolescent was more exposed to the sun. This study shows that caregivers are highly knowledgeable about exposure and sun protection in children/adolescents, and their attitudes follow the general recommendations.

## 1. Introduction

Ultraviolet (UV) radiation is part of the electromagnetic spectrum emitted by the sun [1]. While UV-C rays are absorbed by atmospheric ozone, most UV-A radiation and about 10% of UV-B rays reach the Earth’s surface [1,2]. Small amounts of UV radiation are essential for vitamin D production, but excessive exposure can result in acute and chronic effects on the skin, eyes, and immune system [1,3,4].

According to the World Health Organization (WHO), four out of five cases of skin cancer can be avoided, as the damage caused by UV rays is preventable [3].

Excessive exposure to UV radiation during the first decades of life is related to skin photoaging and the development of skin cancer [1,2,5,6]. Several studies have revealed an association between childhood sunburn and the subsequent risk of melanoma [1,2,5,6,7,8]. Exposure to intermittent but intense UV radiation, especially in childhood, is vital in the pathogenesis of melanoma and basal cell carcinoma. In contrast, cumulative exposure is more important for the development of squamous cell carcinoma [2,9].

Data from the 2018 National Oncological Registry of Portugal point to approximately 9.80 new cases per year of malignant skin melanoma per 100,000 habitants and 984 new cases of melanoma skin cancer [10].

It is estimated that 50 to 80% of the damage induced by sun exposure occurs in childhood and adolescence [11]. Therefore, reducing the levels of this exposure in pediatric age may have a more significant impact on the incidence of skin cancer than in adulthood [6,12,13,14]. Thus, it is essential to optimize caregivers’ knowledge and early implementation of good photoprotection habits in childhood to prevent and reduce skin cancer [12,15,16,17].

The present study aimed to characterize the knowledge and attitudes of caregivers regarding exposure and sun protection of children and adolescents and identify possible relationships between these two factors. The study’s significance is to understand the caregiver’s perspective on exposure and sun protection in children and adolescents and alert for the importance of elucidating caregivers who do not follow the general recommendations regarding the appropriate sun exposure and protection methods. In addition, considering that excessive sun exposure and sunburns during youth are related to the development of melanoma cancer [7,18], this study highlights the importance of adopting preventive sun exposure strategies in a dependent population on their parental knowledge regarding exposure and sun protection.

## 2. Materials and Methods

### 2.1. Study Design and Sample

An observational, analytical, and cross-sectional study was carried out between May and October by conducting a confidential questionnaire directed at a convenience sample of caregivers of children and adolescents (from 0 to 18 years of age). The caregivers resorted to the External Consultation, Inpatient Service, and the Pediatric Emergency Service of Hospital de Santo André in Leiria, Portugal, and consultations at the Cidade do Lis Family Health Unit in Leiria, Portugal, during the defined period. The questionnaire was distributed by technical assistants blinded to the study group while the caregivers were in the waiting room for care. Participation was voluntary and anonymous, and none of the data collected allowed the participant’s identification. All participants provided informed consent. The study was conducted according to the ethical standards of Hospital de Santo André, and Cidade do Lis Family Health Unit. As the present survey research involved independent and competent adults and there were no risks of physical and psychological harm, ethical oversight from an Ethical Review Board was not required [19].

### 2.2. Questionnaire

The questionnaire was elaborated from a literature review and similar studies conducted in Portugal [15,16] and was confidential and self-administered by caregivers. The questions focused on the knowledge and attitudes of caregivers regarding exposure and sun protection of children and adolescents. Questions were asked with only one answer, multiple-choice, and one section included several statements about the caregiver’s knowledge regarding exposure and sun protection in children and adolescents. In the latter, the caregivers rated each statement according to the degree of agreement, using the Likert Scale from 1 (“completely disagree”) to 5 (“completely agree”). To quantify the participant’s knowledge, we conducted a similar set of procedures described in previous research [20]. Firstly, we coded the answers as 1 to 5, where low scores represented the incorrect answer and high scores the correct answer. Secondly, we calculated the total knowledge score as the sum of the ratings in the fifteen statements, generating a score between 15 to 75. A higher score indicated a greater knowledge regarding exposure and sun protection. Finally, using 50% and 75% as cutoff points, we classified the participants as having low knowledge for scores ranging from 15 to 44, average knowledge for scores ranging from 45 to 59, and high knowledge for scores ranging from 60 to 75.

### 2.3. Statistical Analysis

The data obtained were coded in Microsoft Office Excel^®^ and later analyzed in SPSS v27.0^®^. To analyze the internal consistency of the caregiver’s knowledge section in the questionnaire, we calculated Cronbach’s alpha, where values between 0.50–0.69 were considered acceptable, and values between 0.70–0.90 indicated strong internal consistency [21,22]. Descriptive statistics were used to observe the percentage distribution of the sample about nominal and ordinal categories and their absolute frequency. The data referring to the scalar categories were presented as mean ± standard deviation. The Chi-square test was used to compare proportions, and the statistical significance was set at *p* < 0.05.

## 3. Results

### 3.1. Internal Consistency of the Caregiver’s Knowledge Section

The Cronbach’s alpha score of the caregiver’s knowledge section was 0.57, thus suggesting an acceptable internal consistency.

### 3.2. General Results

**Figure 1:**
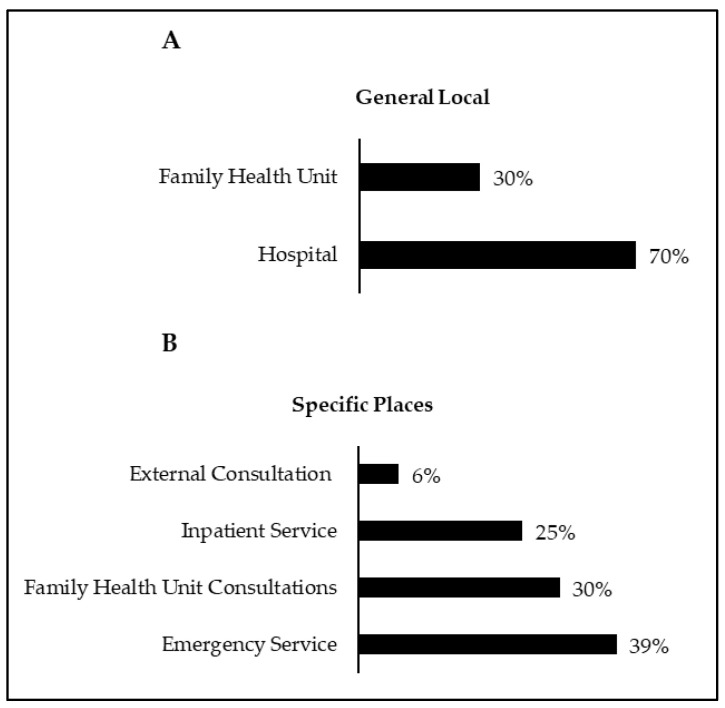
General locations for applying the questionnaire (**A**); specific places of application of the questionnaire (**B**).

Two hundred and twenty questionnaires were delivered, and 198 responses were obtained, corresponding to a participation rate of 90%. Regarding the general locations of questionnaire administration, 30.3% were administered in Cidade do Lis Family Health Unit, and 69.7% in the Pediatric Emergency Service of Hospital de Santo André (Figure 1A). As for the specific locations of questionnaire administration, 38.9% were administered at the emergency service, 30.3% at consultations in the Cidade do Lis Family Health Unit, 24.7% in the inpatient service, and 6.1% in the external consultation (Figure 1B).

### 3.3. Characterization of the Caregiver and the Child/Adolescent

**Figure 2:**
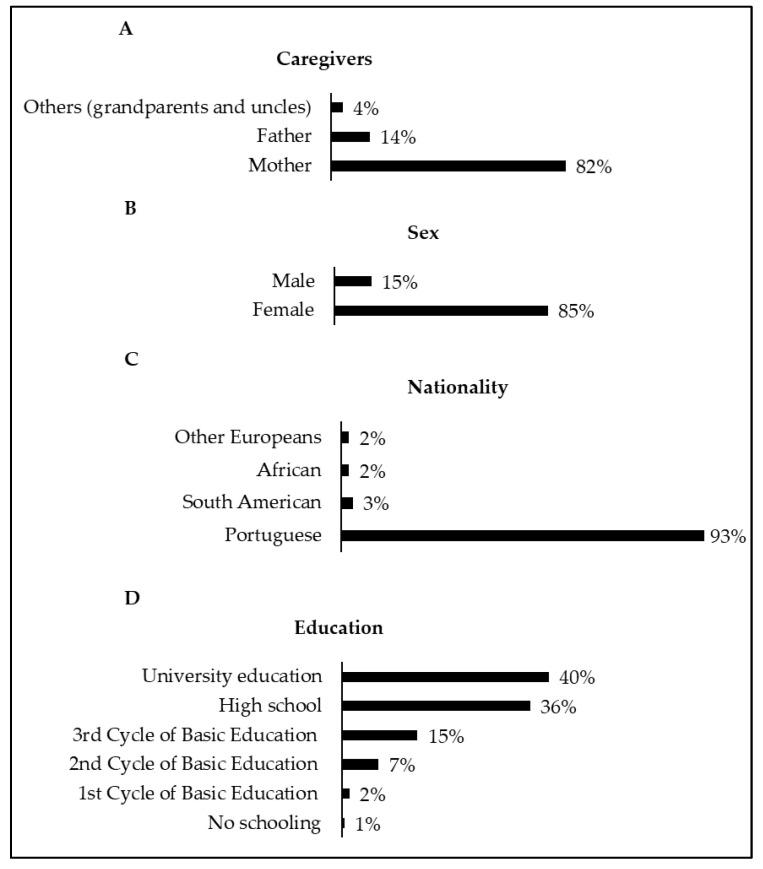
Caregivers who answered the questionnaire (**A**); sex of caregivers (**B**); nationality of caregivers (**C**); caregivers’ education (**D**).

Figure 2 shows that of the 198 questionnaires collected, 96% were answered by parents and 4% by other caregivers (grandparents and uncles). The mean age of the caregivers was 38.5 ± 8.0 years old (range: 19–73 years) and most were female (85%). In addition, 93% of the responders were of Portuguese nationality, 3% of South American origin, 2% from African countries, and 2% from other European nationalities. Regarding the level of education, most completed either University education (40%) or high school (36%), while the rest finished the 3rd cycle of basic education (15%), 2nd cycle of basic education (7%), and 1st cycle of basic education (1.5%). Only one caregiver (0.5%) reported having no level of education. The average age of the children/adolescents was 6.5 ± 5.1 years old (range: 1 month to 17 years old), with 52% female and 48% male.

### 3.4. Caregivers’ Knowledge Regarding Exposure and Sun Protection in Children and Adolescents

Table 1 shows the results obtained on caregivers’ knowledge regarding exposure and sun protection of children and adolescents. A high percentage of caregivers answered correctly in most sentences, except the following ones: “5. The smaller the shade, the more dangerous the sun”, “13. The higher the altitude, the more easily you can get sunburned”, and “15. The protective effectiveness starts right after the application of the sunscreen”. The average total knowledge score of caregivers was 61.4 ± 6.0 (range: 44 to 74). Therefore, on average, the participants were classified as having high knowledge regarding exposure and sun protection in children and adolescents.

**Figure 3:**
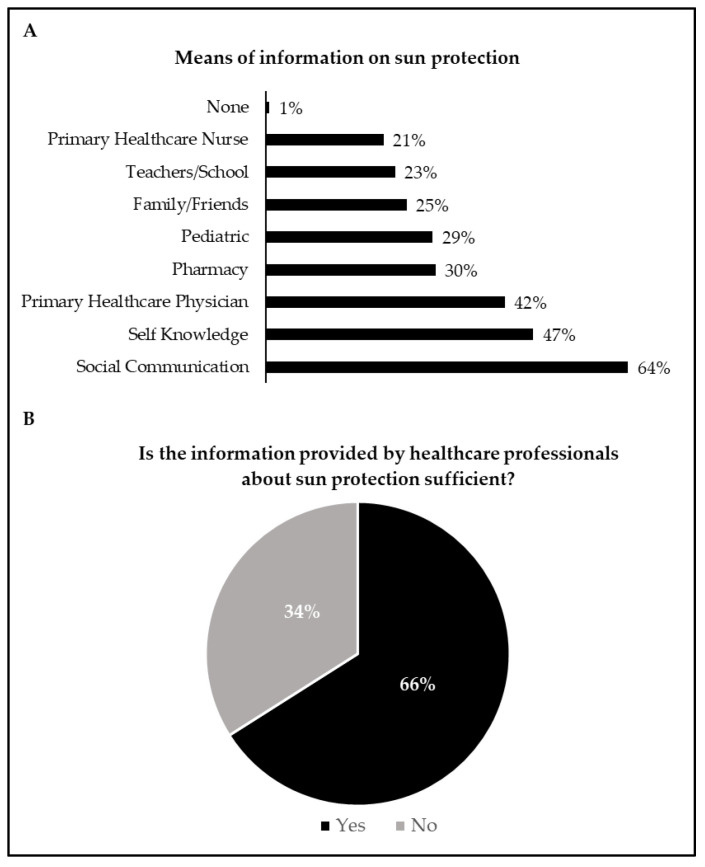
Means of information on sun protection (**A**); information provided by healthcare professionals (**B**). In Figure 3A, the sum of the proportions exceeds 100% because multiple-choice answers were allowed.

Regarding the means where caregivers obtain more information about sun protection and the information provided by health professionals on this topic, the results are shown in Figure 3. In general, social communication was considered the means to acquire more information about sun protection, followed by self-knowledge and primary healthcare physicians. When asked if the information provided by healthcare professionals about sun protection was sufficient, 66% answered “yes”.

### 3.5. Attitudes of Caregivers Regarding Exposure and Sun Protection of Children and Adolescents

**Figure 4:**
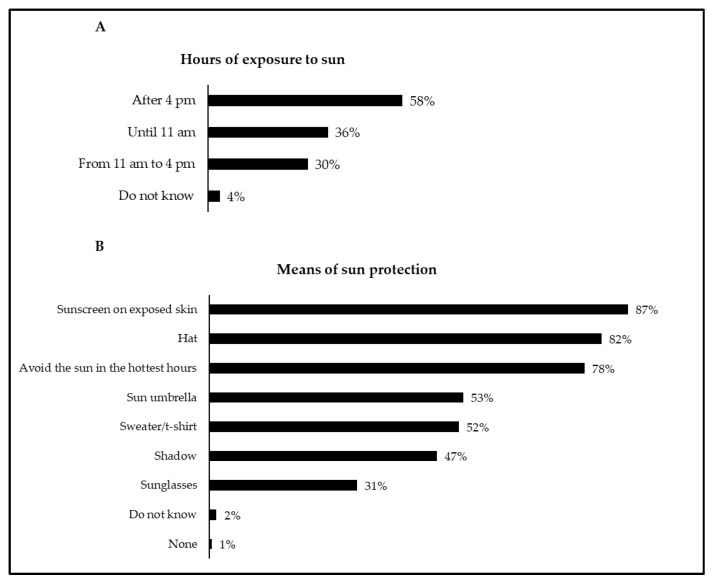
Hours of exposure to the sun (**A**) and means of sun protection (**B**) in the child/adolescent. In both figures, the sum of the proportions exceeds 100% because multiple-choice answers were allowed.

Figure 4 shows the results related to when (time) the child/adolescent was usually more exposed to the sun and the most used means of sun protection. In general, the child/adolescent was more exposed to the sun after 4:00 p.m., and the most selected means of sun protection were sunscreen on exposed skin, hats, and avoiding the sun in the hottest hours.

**Figure 5:**
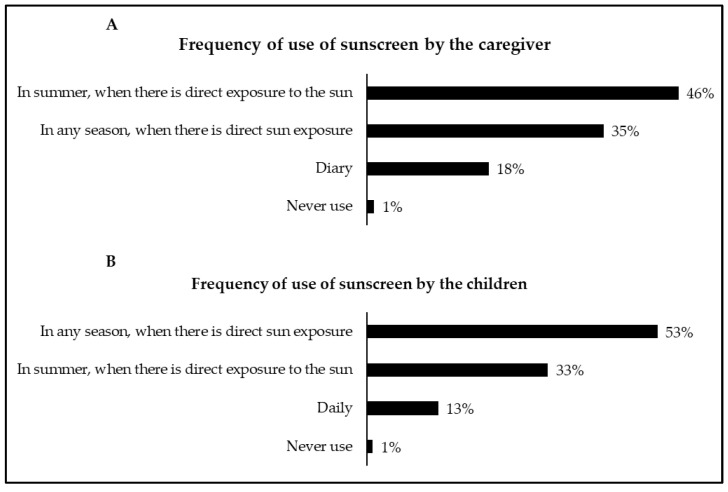
Frequency of use of sunscreen by the caregiver (**A**) and the child/adolescent (**B**).

Figure 5 shows the results related to the frequency of use of sunscreen in the caregiver and child/adolescent. Caregivers reported that they frequently used sunscreen in summer when there was direct exposure to the sun. In addition, they reported that the child/adolescent frequently used sunscreen in any season when they were exposed to the sun.

**Figure 6:**
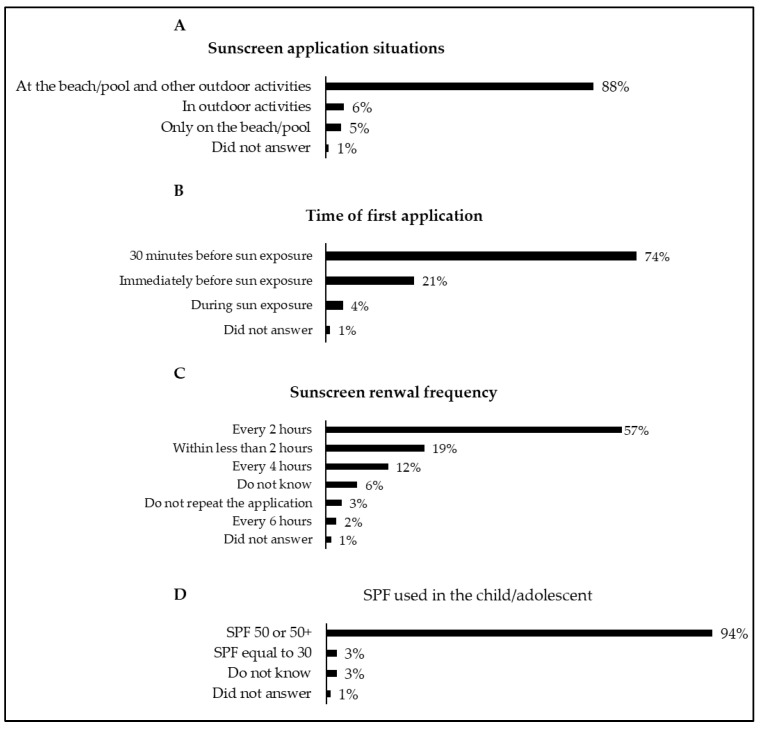
Sunscreen application situations (**A**), time of first application (**B**), sunscreen renewal frequency (**C**), and sun protection factor (SPF) used in the child/adolescent (**D**).

Figure 6 shows the data associated with the sunscreen application situations, the time of first application, the sunscreen renewal frequency, and the sun protection factor (SPF) used by the children/adolescents. Overall, 57% of caregivers reported a renewal sunscreen in the child/adolescent every 2 h, and 94% reported that the child/adolescent used an SPF ≥ 50.

In the last 12 months, it was reported that 90% of children/adolescents did not suffer sunburn after sun exposure, while 8% suffered only one sunburn. In addition, 2% of caregivers answered that they did not know if the children/adolescents had suffered a sunburn in the last 12 months. Finally, 88% of caregivers answered that there was no case of skin cancer in the family, while 5% reported a case, and 7% said they did not know.

### 3.6. Educational Level vs. Caregivers’ Knowledge Regarding Exposure and Sun Protection in Children and Adolescents

There was a significant relationship (*p* < 0.001) between self-knowledge about sun protection and the total knowledge score with education level. In general, caregivers who reported not having self-knowledge about sun protection and presented lower total knowledge scores were those with a lower level of education or even none. In addition, most caregivers who agreed with the following statements presented a lower level of education or no level of education: “3. There is no need to use sun protection measures during the winter months”; “4. At the beach and pool, it is enough to put on sunscreen once”; “7. The proper time to apply the protector is on arrival at the beach”; “10. It is only necessary to use sunscreen at the beach or pool”; and “11. People with dark skin do not need to take special care when they are in the sun”.

### 3.7. Knowledge vs. Attitudes of Caregivers Regarding Exposure and Sun Protection in Children and Adolescents

There was a significant association (*p* < 0.05) between the statement “3. There is no need to use sun protection measures during the winter months” and the frequency of use of sunscreen by the caregiver. Although 83% of caregivers disagreed with the statement, 46% (91/198) reported that they applied sunscreen only in the summer, when there was direct exposure to the sun, 35% (69/198) in any season, when there was direct sun exposure, 18% (36/198) daily and 1% (2/198) never applied. Likewise, there was a significant association (*p* < 0.05) between the statement “3. There is no need to use sun protection measures during the winter months” and the frequency of use of sunscreen in the child/adolescent. Of the 83% of caregivers who disagreed, 53% reported that the child used sunscreen in any season when directly exposed to the sun, 33% reported that the child used sunscreen only in the summer when there was direct exposure to the sun, 13% reported that the child applied daily, and 1% stated that children never applied sunscreen.

Concerning the statement “7. The proper time to apply the protector is on arrival at the beach,” and the moment when the caregiver made the first application of sunscreen to the child/adolescent, there was a significant association (*p* < 0.001). Of the 82% that disagreed with the statement, 74% reported that they applied sunscreen for the first time to the child/adolescent 30 min before sun exposure. Finally, there was a significant association (*p* < 0.05) between the statement “9. Avoid sun exposure between 11:00 a.m. and 4:00 p.m.” and the time when the child/adolescent was usually more exposed to the sun. Of the 187 respondents who agreed with the statement, 71% reported that from 11:00 a.m. to 4:00 p.m., children/adolescents were not exposed to the sun.

## 4. Discussion

### 4.1. General Findings

In the current research, we aimed to characterize the knowledge and attitudes of caregivers regarding exposure and sun protection of children and adolescents and establish relationships between them. Our results showed that, on average, caregivers presented high knowledge regarding exposure and sun protection in children and adolescents, which is in line with the results reported in previous studies conducted in European countries [23,24,25,26,27]. In addition, our results also demonstrated that most caregivers’ attitudes followed the national and international guidelines about skin cancer prevention. In this sense, these results are globally optimistic since previous research found that caregivers with a high level of sun protection behavior are less likely to report being sunburnt, and consequently, their children are more likely to have a high level of sun protection behavior and sunscreen vigilance [28].

### 4.2. Caregivers’ Knowledge Regarding Exposure and Sun Protection in Children and Adolescents

There was a high percentage of correct answers in practically all statements, which generated, on average, a high level of knowledge. However, for the statements “5. The smaller the shade, the more dangerous the sun”, “13. The higher the altitude, the more easily you can get sunburned”, and “15. The protective effectiveness starts right after the application of the sunscreen”, there was an inconsistency about the correct answer according to the scientific evidence [2]. Thus, these results indicate the need for additional clarification on these specific issues by healthcare professionals during medical consultations. In fact, it is essential to notice that when caregivers were asked if the information provided by healthcare professionals about sun protection was enough, only 64% provided an affirmative answer. Therefore, these data reinforce the importance of healthcare organizations promoting regular health education campaigns and skin cancer prevention programs due to their effectiveness in improving sun protection knowledge among caregivers [24,29,30,31,32].

Our study found an association between self-knowledge and the total knowledge score regarding exposure and sun protection with the education level. Moreover, there was an association between the education level with the following statements: “3. There is no need to use sun protection measures during the winter months”; “4. At the beach and pool, it is enough to put on sunscreen once”; “7. The proper time to apply the protector is on arrival at the beach”; “10. It is only necessary to use sunscreen at the beach or pool”; and “11. People with dark skin do not need to take special care when they are in the sun”. Therefore, these results suggest that the higher the caregiver’s education level, the higher the caregiver’s knowledge regarding exposure and sun protection, and vice-versa. Consequently, healthcare professionals should be more careful when disseminating information about skin cancer prevention to caregivers with lower levels of education. In this case, regular monitoring can be a valuable strategy to update their knowledge and, simultaneously, control their sun protection habits because a knowledge deficit of UV and risk of melanoma is associated with an increased risk of sunburn [33].

### 4.3. Means of Information on Sun Protection

Our findings showed that social communication is the primary means for caregivers obtaining exposure and sun protection knowledge. Similarly, in another Portuguese study that aimed to assess the knowledge and attitudes of caregivers about sun protection for children/adolescents, the authors also found that social communication was the means through which caregivers obtained more information about sun protection [16]. Interestingly, a German study found a positive association between the caregiver’s knowledge about skin cancer prevention and print media, audio-visual or personal channels of information [34], reinforcing the importance of these means to disseminate knowledge on this topic. Nevertheless, the authors also observed a negative relationship between internet use and caregivers’ knowledge, which might be associated with the spread of misleading information on the web [34]. In fact, as recently documented, there are significant deficits in the internet’s quality of information for skin cancer prevention [35], suggesting that this source of information should be carefully used. For example, a recent study found that Google was the most common tool parents used to search for pediatric dermatology concerns [36]. Notably, the authors found that Google searches may lead to changes in seeking medical care in pediatric dermatology [36]. Therefore, it is essential that pediatrics carefully address the inappropriate and excessive use of online searches for health information during medical consultants to ease parents’ rising concerns [36].

### 4.4. Caregivers’ Attitudes Regarding Exposure and Sun Protection in Children and Adolescents

Concerning caregivers’ attitudes towards exposure and sun protection of children and adolescents, our data found an association between the statement “9. Avoid sun exposure between 11:00 a.m. and 4:00 p.m.” and the time when the child/adolescent was usually more exposed to the sun, namely from 11:00 a.m. to 4:00 p.m. In general, most caregivers reported that from 11:00 a.m. to 4:00 p.m., children/adolescents were not exposed to the sun, which is in line with the recommended guidelines [2,9,37,38] and previous studies conducted in Portugal [15,16]. As for the means of sun protection used by children and adolescents, most caregivers selected the options “avoiding the sun in the hottest hours”, “hat”, and “applying sunscreen to exposed skin”. These results align with previous national and international studies that also found sunscreen and hats as the most common ways of sun protection in children [6,15,16,17,26,39]. Contrary, our results showed that sunglasses were the means of sun protection less used by children and adolescents. Similarly, previous studies also reported low overall use of sunglasses by children and adolescents as a sun protection measure [16,26,39,40]. This generalized occurrence might be related to sunglasses having not yet been established by the scientific literature as an essential sun protection measure for children [39]. Nevertheless, several studies conducted with adults have observed that high levels of sun exposure are a risk factor for cataracts [41,42], thus suggesting that the use of sunglasses might be a protective way against UV radiation.

Still, on caregivers’ attitudes, our results showed that most use sunscreen only in summer when there is direct sun exposure, followed by a lower percentage that uses it in any season when there is direct sun exposure. Interestingly, these data were associated with the statement “3. There is no need to use sun protection measures during the winter months”. Thus, although most disagreed with the statement, the attitudes of around half of caregivers do not represent what they reported. In addition, the attitudes adopted do not follow the general recommendations, which state that sunscreen should be used in any season, whenever there is direct exposure to the sun [2,37]. Thus, greater dissemination of this type of information by healthcare professionals to caregivers is needed. On the contrary, when the sunscreen user was the child/adolescent, half of the caregivers indicated that they applied sunscreen in any season when there was direct exposure to the sun, while one-third reported only in summer when there was direct exposure to the sun. Likewise, the data were associated with the statement “3. There is no need to use sun protection measures during the winter months”. Thus, although most disagreed with the statement, one-third still apply sunscreen to the child only in the summer when there is direct exposure to the sun, which contradicts the general recommendations [2,37]. Therefore, as previously observed, although caregivers generally present a broad knowledge regarding sun protection recommendations, they usually adopt a range of sun-protective measures for their child depending on the time of year [43]. Therefore, given that caregivers seemed to adopt different sun protection behaviors throughout the year, more regular health education campaigns should be promoted by health organizations.

About 88% of caregivers applied sunscreen to children/adolescents at the beach/pool and outdoor activities, and 74% applied sunscreen 30 min before sun exposure. Data on the moment of sunscreen application on children showed an association with the statement “7. The proper time to apply the protector is on arrival at the beach”, as 82% of caregivers disagreed with the statement. Therefore, there is a degree of agreement between the caregivers’ attitudes towards sunscreen application on children and their knowledge. Still, on the application of sunscreen, 57% renewed the application of the sunscreen every 2 h in the child/adolescent, and 94% used an SPF ≥ 50. These data reveal that caregivers’ attitudes align with national [37] and international [2,9,38] recommendations, which suggest applying sunscreen about 15 to 30 min before sun exposure to allow for adequate absorption and renewing the application every 2 h using an SPF ≥ 30. Interestingly, a recent UV photography study observed an immediate protective effect of sunscreen and suggested shorter waiting times than the classical 30 min until sun exposure [44]. However, it is essential to note that a recent review recommended waiting 15–30 min until sun exposure because the grade of recommendation on this topic was weak [45].

Although we did not find significant associations between sunburn in the last year and caregivers’ knowledge and attitudes, it is essential to note that our results indicated that 8% of children/adolescents experienced one sunburn in the prior 12 months. These results should alert clinicians for the importance of clarifying caregivers on the appropriate use of sun protection, considering the strong correlations between sunburn during childhood and the development of melanoma in adult life [7,18].

### 4.5. Study Limitations

The current study presents some limitations that should be addressed. Firstly, the use of a convenience sample may not represent the knowledge and attitudes of the general population of Portuguese caregivers regarding exposure and sun protection in children and adolescents. Although studies conducted in different regions of Portugal [15,16] observed similar findings regarding the knowledge and attitudes of caregivers on this topic, it is essential to note that most caregivers also completed either university education or high school. Therefore, although these results might suggest that Portuguese caregivers are aware of the sun protection recommendations, caution should be taken when generalizing these findings to less-educated caregivers. A second study limitation might be related to applying a self-administered questionnaire, which might have created difficulty for some of the caregivers to understand some of the questions. In addition, the formulation of the questions in the multiple-choice format might have induced some answers that did not fully correspond to the actual practice. A third limitation is that caregivers may have overreported their sun-protective attitudes to reflect socially desirable behavior instead of reporting precise information about their actual behaviors, thus leading to a potential source of information bias [26]. Finally, as reported in similar research studies [26,36], recall and memory bias should also be recognized as limitations since at least one question was related to past events. Nevertheless, the relevance of this study is highlighted given the few scientific studies in Portugal on this topic.

## 5. Conclusions

In summary, the current research demonstrates that, in general, caregivers are highly knowledgeable about exposure and sun protection in children/adolescents, and their attitudes follow the national and international guidelines. During Child and Youth Health surveillance, interventions are carried out to achieve objectives and continuously obtain health gains in this population. One of the main goals is to prevent risks arising from inadequate sun exposure [46]. Thus, it is essential that healthcare professionals, mainly primary healthcare physicians, nurses, and pediatricians, encourage parental education through the elucidation of anticipatory care during surveillance consultations and the availability of information leaflets to understand the local reality for a more practical application. In this sense, this study contributes to better understanding the caregiver’s perspective on exposure and sun protection, their knowledge and attitudes, and alerts to the need to invest in training parents and healthcare professionals about this relevant theme.

## Figures and Tables

**Table 1:** Results in percentage (%) on caregivers’ knowledge regarding exposure and sun protection of children and adolescents.

Sentence	CD	D	NAND	A	CA
1. Excessive sun exposure carries health risks such as sunburn, premature skin aging, skin cancer, or eye problems.	0.5%	0.5%	1%	14%	84%
2. The risk of developing skin cancer is related to the amount of ultraviolet radiation a person is exposed to during their lifetime, especially during childhood.	1%	3%	11%	42%	43%
3. There is no need to use sun protection measures during the winter months.	33%	50%	10%	5%	2%
4. At the beach and pool, it is enough to put on sunscreen once.	59%	29%	3%	5%	4%
5. The smaller the shadow, the more dangerous the sun.	6%	17%	26%	28%	23%
6. There is no danger of being exposed to the sun longer if you use sunscreen.	32%	51%	9%	6%	2%
7. The proper time to apply the protector is on arrival at the beach.	34%	48%	4%	11%	3%
8. It is possible to suffer a sunburn on a cloudy day.	1%	3%	3%	35%	58%
9. Avoid sun exposure between 11:00 a.m. and 4:00 a.m.	3%	1%	2%	31%	63%
10. It is only necessary to use sunscreen at the beach or pool.	48%	45%	4%	1%	2%
11. People with dark skin do not need to take special care when they are in the sun.	50%	46%	1.5%	2%	0.5%
12. The tan protects you from additional sunburn.	41%	49%	8%	2%	0%
13. The higher the altitude, the more easily you can get sunburned.	4%	20%	33%	30%	13%
14. Being in the shade of a sun hat or awning is enough to avoid getting sunburned.	24%	54%	13%	8%	1%
15. The protective effectiveness starts right after the application of the sunscreen.	11%	35%	17%	34%	3%

CD: completely disagree; D: disagree; NAND: neither agree nor disagree; A: agree; CA: completely agree.

## Data Availability

Not applicable.
